# Dysfunction of Organic Anion Transporting Polypeptide 1a1 Alters Intestinal Bacteria and Bile Acid Metabolism in Mice

**DOI:** 10.1371/journal.pone.0034522

**Published:** 2012-04-04

**Authors:** Youcai Zhang, Pallavi B. Limaye, Lois D. Lehman-McKeeman, Curtis D. Klaassen

**Affiliations:** 1 Department of Pharmacology, Toxicology and Therapeutics, University of Kansas Medical Center, Kansas City, Kansas, United States of America; 2 Pharmaceutical Candidate Optimization, Bristol-Myers Squibb Co., Princeton, New Jersey, United States of America; Charité, Campus Benjamin Franklin, Germany

## Abstract

Organic anion transporting polypeptide 1a1 (Oatp1a1) is predominantly expressed in liver and is able to transport bile acids (BAs) *in vitro*. Male Oatp1a1-null mice have increased concentrations of taurodeoxycholic acid (TDCA), a secondary BA generated by intestinal bacteria, in both serum and livers. Therefore, in the present study, BA concentrations and intestinal bacteria in wild-type (WT) and Oatp1a1-null mice were quantified to investigate whether the increase of secondary BAs in Oatp1a1-null mice is due to alterations in intestinal bacteria. The data demonstrate that Oatp1a1-null mice : (1) have similar bile flow and BA concentrations in bile as WT mice; (2) have a markedly different BA composition in the intestinal contents, with a decrease in conjugated BAs and an increase in unconjugated BAs; (3) have BAs in the feces that are more deconjugated, desulfated, 7-dehydroxylated, 3-epimerized, and oxidized, but less 7-epimerized; (4) have 10-fold more bacteria in the small intestine, and 2-fold more bacteria in the large intestine which is majorly due to a 200% increase in *Bacteroides* and a 30% reduction in *Firmicutes*; and (5) have a different urinary excretion of bacteria-related metabolites than WT mice. In conclusion, the present study for the first time established that lack of a liver transporter (Oatp1a1) markedly alters the intestinal environment in mice, namely the bacteria composition.

## Introduction

**Figure 1:**
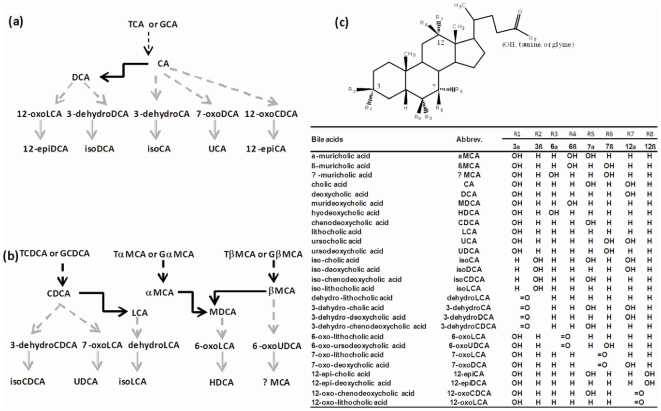
Bacteria-mediated BA metabolism. (a) Deconjugation, dehydroxylation, oxidation, and epimerization of cholic acid (CA) in the intestine of mice. Black dotted arrows represent for deconjugation, black solid arrows represent for 7-dehydroxylation, grey dotted arrows represent for oxidation, grey solid arrows represent for epimerization; (b) Deconjugation, dehydroxylation, oxidation, and epimerization of chenodeoxycholic acid (CDCA) and muricholic acid (MCA) in the intestine of mice. Black dotted arrows represent for deconjugation, black solid arrows represent for 7-dehydroxylation, grey dotted arrows represent for oxidation, grey solid arrows represent for epimerization; (c) Structures and abbreviations of various BAs. TCA and GCA stand for taurocholic acid and glycocholic acid, respectively.

Bile acids (BAs) are synthesized from cholesterol, conjugated with taurine or glycine in liver, secreted into bile, and then delivered to the intestine. The majority of BAs are absorbed from the intestine and return to the liver, which together is known as the enterohepatic circulation of BAs [1], [2]. In rodents, primary BAs, which are synthesized in liver include cholic acid (CA), chenodeoxycholic acid (CDCA), α-muricholic acid (αMCA), and βMCA. Secondary BAs are formed from primary BAs by bacterial enzymes in the intestine. The major secondary BAs in rodents are deoxycholic acid (DCA), lithocholic acid (LCA), and murideoxycholic acid (MDCA), which are the 7-dehydroxylation products of CA, CDCA, and α/βMCA, respectively. The bacterial metabolism of BAs as well as the structures and abbreviations of various BAs are shown as Fig. 1.

The intestinal microbiota in both humans and mice consist mainly of *Bacteroidetes* and *Firmicutes* phyla [3], [4]. More than 95% of the *Firmicutes* are members of the *Clostridia* class. Non-culture based techniques that rely on 16 S rRNA gene sequences are increasingly being used for the identification and classification of bacterial species [4], [5].

Bacterial bile salt hydrolases (BSHs) catalyze BA deconjugation, and hydroxysteroid dehydrogenases (HSDHs) catalyze BA oxidation and epimerization [6]. Only a few species of intestinal bacteria have been examined to determine their ability to metabolize BAs. For example, *Clostridium* (*C.*) *absonum* (expresses both 7α- and 7β-HSDHs, and is able to epimerize CDCA to ursodeoxycholic acid (UDCA) [7], [8]. *C. perfringens* expresses both BSH [9] and HSDHs [10]. *C. scindens* expresses both 3α- and 7α-HSDHs, and also has high BA 7α-dehydroxylation activity [11]. BSHs are also expressed in Lactobacillus *(La.) acidophilus*, *La. reuteri*, *and Bacteroides (Ba.) vulgatus* [12], [13], [14]. *Ba. distasonis* has BA 7β-dehydroxylation activity [15].

Organic anion transporting polypeptides (human: OATPs; rodents: Oatps) mediate the transport of a variety of structurally diverse endogenous compounds (such as prostaglandins, thyroid hormones, conjugated steroids, and BAs) and xenobiotics (such as anticancer drugs, antibiotics, cardiac glycosides, and some peptides) [16]. In mice, Oatp1a1, 1a4, and 1b2 are predominantly expressed in liver [17]. Oatp1a1 has been shown to transport BAs, such as taurocholic acid (TCA) *in vitro* [18]. Thus, Oatp1a1 is thought to transport BAs *in vivo* from blood into liver. In a previous study, Oatp1a1-null mice were found to have increased secondary BAs (DCA and TDCA) in serum, a finding that could not be attributed to decreased hepatic uptake, as might be expected in this model [19]. These Oatp1a1-null mice also showed increased urinary excretion of isethionic acid, a taurine metabolite produced by intestinal bacteria [20], suggesting potential perturbations in the intestinal microbiome. Accordingly, we hypothesized that the increase of DCA and TDCA in serum of Oatp1a1-null mice is due to an increase in intestinal bacteria, and therefore the purpose of the present study is to investigate the *in vivo* role of Oatp1a1 in BA metabolism and intestinal bacteria composition.

## Results

### BA Concentrations in Feces

**Figure 2:**
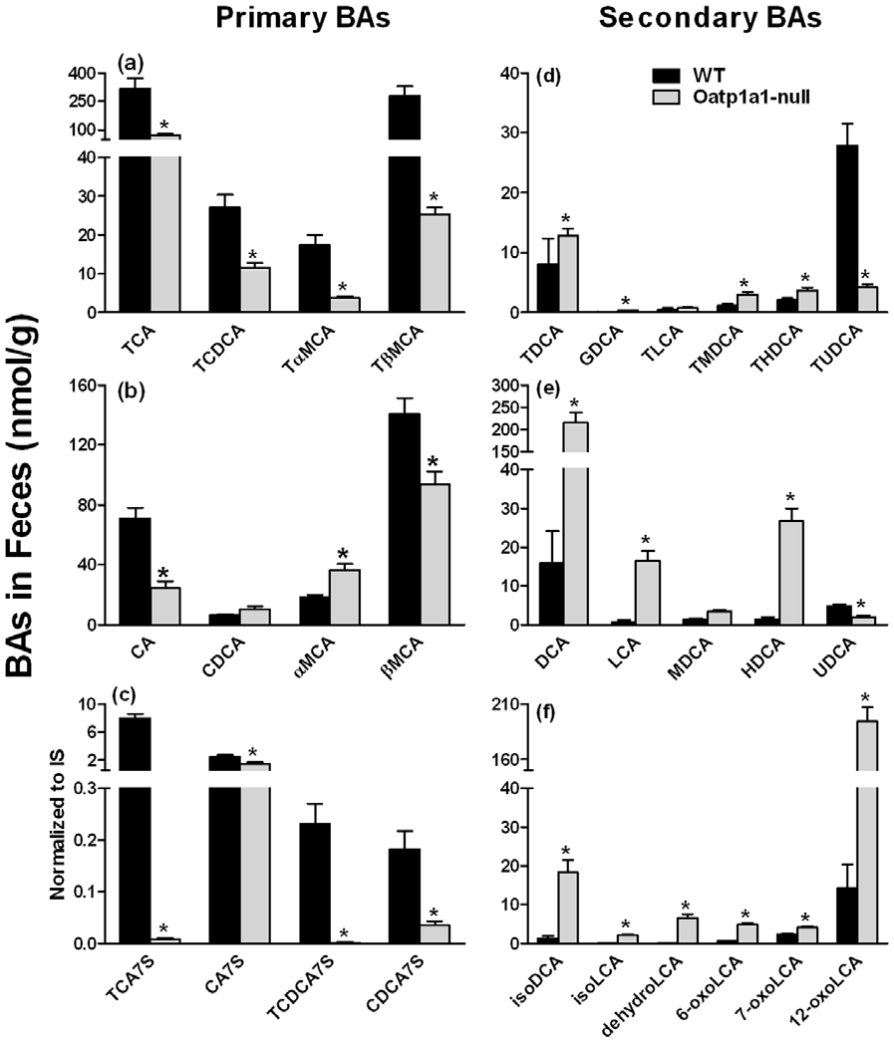
Concentrations of primary and secondary BAs in the feces of WT and Oatp1a1-null mice. Feces were collected from WT and Oatp1a1-null mice for 24 hr and dried under vacuum. The concentrations of primary BAs (a, b, and c) and secondary BAs (d, e, and f) in the feces of male WT and Oatp1a1-null mice (n = 5/group) were analyzed using ultra performance liquid chromatography-tandem mass spectrometry (UPLC-MS/MS) and normalized by fecal dry weight. All data are expressed as mean ± S.E. of five mice in each group. *, statistically significant difference between WT and Oatp1a1-null mice (*p*<0.05).

A previous study demonstrated that secondary BAs including DCA and TDCA, which are produced by intestinal bacteria, were increased in the serum of Oatp1a1-null mice [19]. To investigate whether the increased secondary BAs in serum of Oatp1a1-null mice is due to alterations in intestinal BA metabolism, the BA concentrations in the feces of Oatp1a1-null mice were quantified. Fig. 2a illustrates the concentrations of conjugated primary BAs in feces of mice. Lack of Oatp1a1 markedly decreased TCA (80%), TCDCA (60%), TαMCA (80%), and TβMCA (90%). Fig. 2b illustrates the concentrations of unconjugated primary BAs in feces of mice. Lack of Oatp1a1 decreased CA about 65%, but had no effect on CDCA. Lack of Oatp1a1 increased αMCA about 95%, but decreased βMCA about 30%. BA sulfates can be desulfated by bacterial enzymes in the intestine [21], and in Oatp1a1-null mice, sulfated BAs were markedly decreased. These included TCA-7S and TCDCA-7S, which were virtually non-detectable in the Oatp1a1-null mice, as well as 40% decrease in CA-7S and 80% decrease in CDCA-7S (Fig. 2c). Taken together, lack of Oatp1a1 appeared to alter both the conjugation and sulfation of BAs in the intestine of mice.

Bacteria-mediated 7-dehydroxylation is a major modification of BAs in the intestine. DCA and LCA are 7-dehydroxylation products of CA and CDCA, respectively. In mice, MDCA is thought to be produced by 7-dehydroxylation of βMCA, and HDCA is produced by further epimerization of MDCA [22]. Lack of Oatp1a1 increased GDCA (420%), TMDCA (170%), and THDCA (1700%), DCA (1300%), LCA (1900%), and HDCA (1700%) (Fig. 2d and 2e). UDCA is produced by 7-epimerization of CDCA by bacterial enzymes in the intestine [23], and in the absence of Oatp1a1, both TUDCA and UDCA were decreased 75 and 60%, respectively (Fig. 2d and 2e). Therefore, lack of functional Oatp1a1 increased 7-dehydroxylation, but decreased 7-epimerization of BAs in the intestine of mice.

IsoDCA and isoLCA are produced by the 3α/β-epimerization of DCA and LCA, respectively. The concentrations of isoDCA (1300%) and isoLCA (1900%) were increased markedly in feces of Oatp1a1-null mice (Fig. 2f). The concentrations of oxo-BAs, such as dehydroLCA (4300%), 6-oxoLCA (700%), 7-oxoLCA (70%), and 12-oxoLCA (1300%) were also increased markedly in the feces of Oatp1a1-null mice (Fig. 2f). Therefore, lack of Oatp1a1 increased 3-epimerization and oxidation of BAs in the intestine of mice.

### BA Concentrations in Liver and Bile

**Figure 3:**
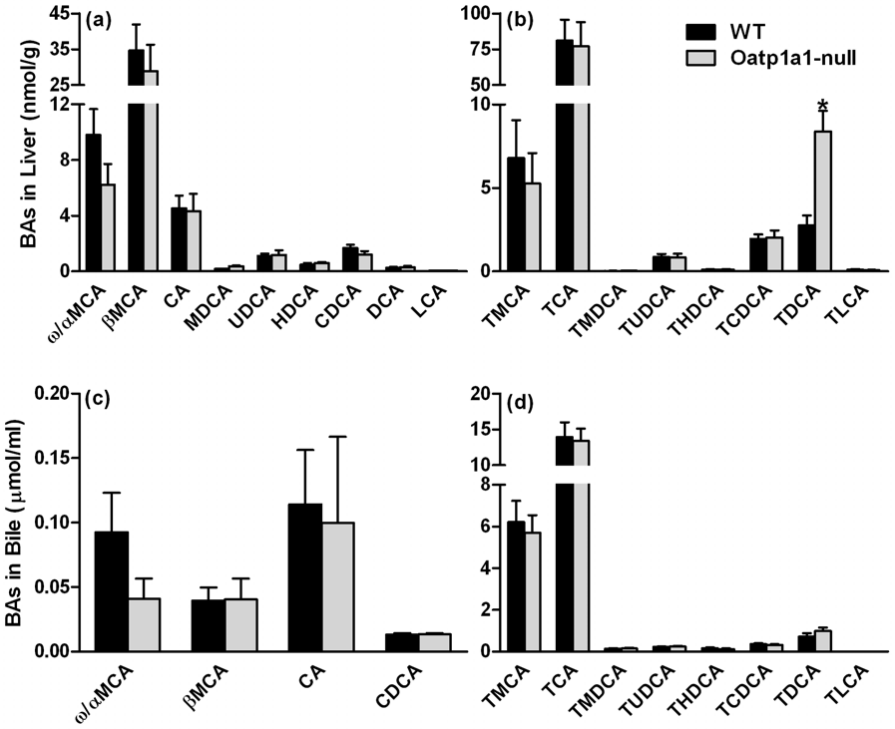
BA concentrations in livers (a and b) and bile (c and d) of mice. BA concentrations in livers and bile of male WT and Oatp1a1-null mice (n = 5/group) were analyzed using ultra performance liquid chromatography-tandem mass spectrometry (UPLC-MS/MS). All data are expressed as mean ± S.E. for five mice in each group. *, statistically significant difference between WT and Oatp1a1-null mice (*p*<0.05).

To investigate whether BA alterations in feces are due to altered hepatic BA metabolism and/or biliary BA excretion, the concentrations of individual BAs in livers and bile of WT and Oatp1a1-null mice were quantified. Lack of Oatp1a1 had little effect on the concentrations of unconjugated BAs in liver (Fig. 3a). Lack of Oatp1a1 also had little effect on most conjugated BAs in liver, except that it increased the concentration of TDCA about 200% (Fig. 3b). In addition, lack of Oatp1a1 had no effects on bile flow (data not shown) or the concentrations of BAs in bile of mice (Fig. 3c and 3d). Taken together, lack of Oatp1a1 increased TDCA concentrations in liver, but had little effect on the composition of BAs in bile.

### BA Composition in the Intestinal Contents

**Figure 4:**
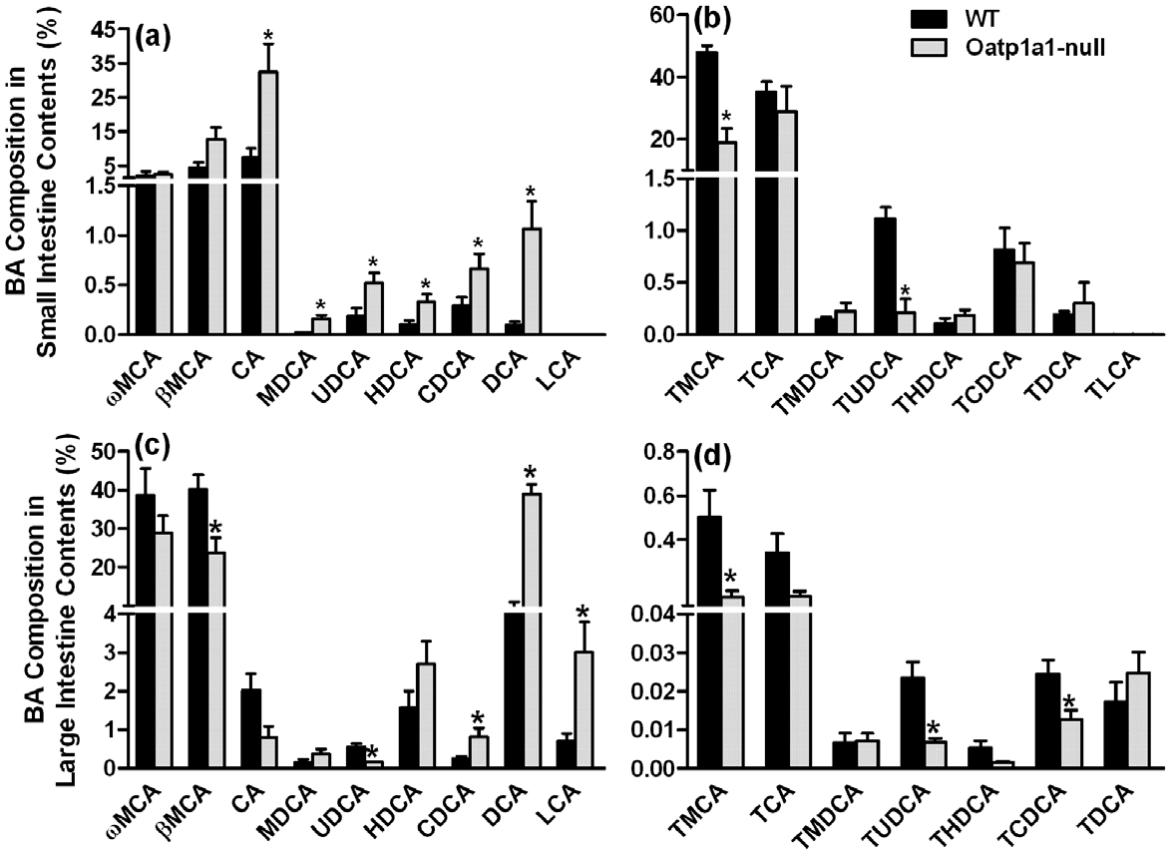
BA composition in the small (a and b) and large (c and d) intestinal contents of mice. BA concentrations in the intestinal contents of male WT and Oatp1a1-null mice (n = 5/group) were analyzed using ultra performance liquid chromatography-tandem mass spectrometry (UPLC-MS/MS). All data are expressed as mean ± S.E. for five mice in each group. *, statistically significant difference between WT and Oatp1a1-null mice (*p*<0.05).

Because the concentrations of BAs in bile are similar between WT and Oatp1a1-null mice, alterations in the fecal BA composition are most likely caused by changes in BA metabolism in the intestinal contents. As shown in Fig. 4a, the major unconjugated BAs in the small intestinal contents of WT mice were ωMCA, βMCA, and CA, whereas in Oatp1a1-null mice, unconjugated BAs were markedly increased including CA (330%), MDCA (1150%), UDCA (170%), HDCA (220%), CDCA (130%), and DCA (1000%). The conjugated BAs were the predominant BAs in the small intestinal contents of WT mice (Fig. 4b). The two major conjugated BAs in WT mice were TMCA and TCA. In contrast in Oatp1a1-null mice, TMCA (60%) and TUDCA (81%) were decreased markedly.

The major BAs in the large intestinal contents of WT mice were unconjugated BAs (Fig. 4). Lack of Oatp1a1 decreased βMCA (40%) and UDCA (69%), but increased CDCA (210%), MDCA (320%), HDCA (150%), DCA (310%), and LCA (320%) (Fig. 4d). Lack of Oatp1a1 decreased TMCA (70%), TUDCA (70%), and TCDCA (50%), but increased TDCA about 280% in the large intestinal contents (Fig. 4d).

### Bacteria in Intestinal Contents

**Figure 5:**
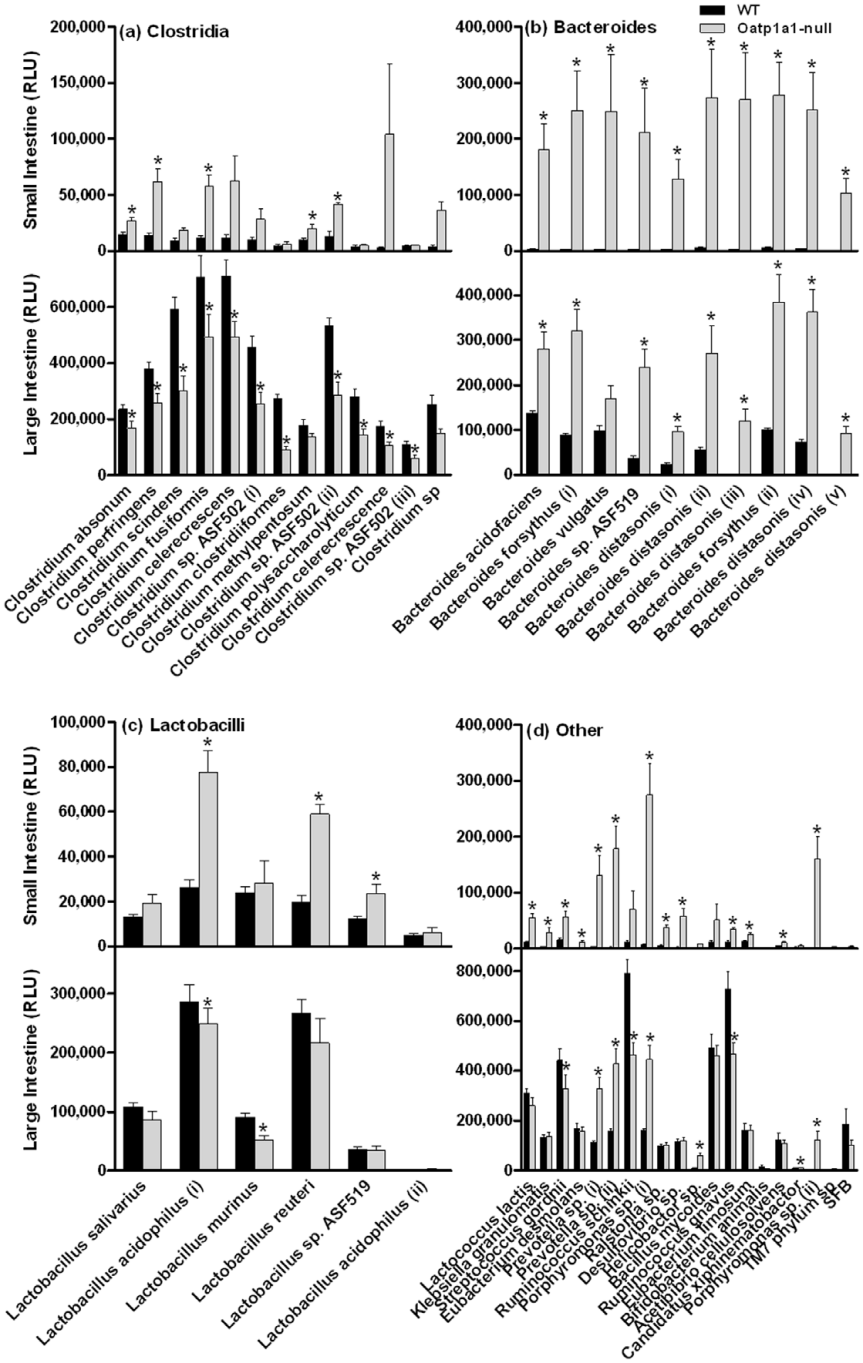
Small and large intestinal bacteria in WT and Oatp1a1-null mice. Clostridia (a), Bacteroides (b), Lactobacilli (c), and other bacteria (d) in the small and large intestinal contents of WT and Oatp1a1-null mice were quantified using a branched DNA assay (Panomics/Affymetrix, Fremont, CA). All data are expressed as mean ± S.E. of five mice in each group. *, statistically significant difference between WT and Oatp1a1-null mice (*p*<0.05).

To investigate whether altered BA metabolism in the intestinal contents of Oatp1a1-null mice is due to alterations in intestinal bacteria, we developed a 16 S rDNA-based assay to quantify bacteria in the intestinal contents of Oatp1a1-null mice. Fig. 5a illustrates the relative amount of *Clostridia* in both small and large intestine of WT and Oatp1a1-null mice. In the small intestine of Oatp1a1-null mice, there was an increase in the following bacteria: *C. absonum* (85%), *C. perfringens* (350%), *C. scindens* (100%), *C. methylpentosum* (110%), and *C. sp. ASF502*(ii) (220%). In contrast, the large intestine of Oatp1a1-null mice showed a decrease in almost all *Clostridia*, including *C. absonum* (30%), *C. perfringens* (30%), *C. sindens* (50%), *C. fusiformis* (30%), *C. celerecrescens* (30%), *C. sp. ASF502* (i, ii and iii) (45%), *C. clostridiiformes* (65%), *C. polysaccharolyticum* (50%), and *C. celerecrescence* (40%).

As shown in Fig. 5b, the small intestine of Oatp1a1-null mice had a 50–100 fold increase in almost all the *Bacteroides*, including *Ba. acidifaciens*, *Ba. forsythus* (i and ii), *Ba. vulgatus*, *Ba. sp. ASF519*, and *Ba. distasonis* (i, ii, iii, iv, and v). In addition, the large intestine of Oatp1a1-null mice also had an increase in *Ba. acidofaciens* (105%), *Ba. forsythus* (ii and ii) (250%), *Ba. sp. ASF519* (540%), *Ba. distasonis* (i, ii, and iv) (300–400%), and *Ba. distasonis* (iii and v) (78–88 fold).

The small intestine of Oatp1a1-null mice had an increase in several *Lactobacilli* (Fig. 5c), such as *La. acidophilus* (190%), *La. reuteri* (200%), and *La.* sp. ASF519 (90%). In contrast, the large intestine of Oatp1a1-null mice had a decrease in *La. acidophilus* (13%) and *La. murinus* (40%).

As shown in Fig. 5d, the small intestine of Oatp1a1-null mice underwent a 100–800% increase in *Lactococcus lactis*, *Klebsiella granulomatis*, *Streptococcus gordnii*, *Eubacterium desmolans*, *Ralstonia* sp., *Eubacterium limosum*, *Acetibibrio cellulosolvens*, and *Ruminococcus gnavus*, as well as 2000–10,000% increase in *Prevotella sp.* (i, and ii), *Porphyromonas sp.*(i), *Desulfovibrio sp.*, and *Porphyromonas sp.*(ii). In contrast, the large intestine of Oatp1a1-null mice underwent a decrease in *Streptococcus gordnii* (25%), *Ruminococcus schinkii* (40%), *Ruminococcus gnavus* (35%), but increased *Prevotella sp.* (i, and ii) (200%), *Porphyromonas sp.* (i) (170%), *Porphyromonas sp.* (ii) (9000%), and *Helicobactor* sp. (600%).

### Fgf15-Fgfr4 Pathway

**Figure 6:**
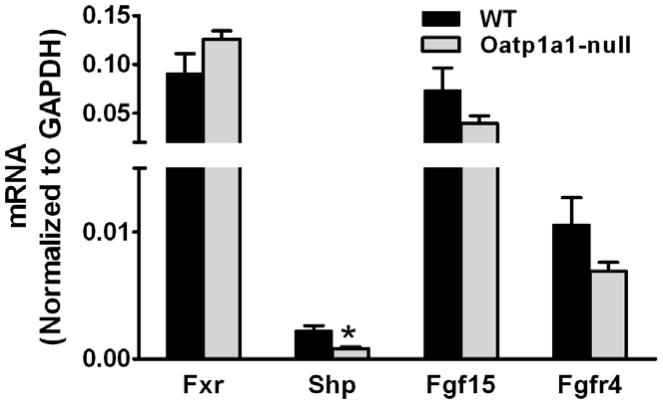
mRNA of Fxr, Shp, and Fgf15 in ilea as well as Fgfr4 in livers of mice. Total RNA from ilea and livers of male WT and Oatp1a1-null mice (n = 5/group) was analyzed using multiplex suspension array. The mRNA of each gene was normalized to GAPDH. All data are expressed as mean ± S.E. of five mice in each group. *, statistically significant difference between WT and Oatp1a1-null mice (*p*<0.05).

Fxr plays a key role in the regulation of BA homeostasis. Fig. 6 illustrates the mRNA expression of Fxr, Shp, and Fgf15 in ilea as well as Fgfr4 in livers of WT and Oatp1a1-null mice. Lack of Oatp1a1 had little effect on ileal Fxr mRNA expression, whereas Oatp1a1-null mice had lower ileal Shp mRNA than did WT mice. In addition, Oatp1a1-null mice tended to have lower mRNA expression of Fgf15 in ilea and Fgfr4 in livers than WT mice, although this difference was not statistically significant.

### Urinary Metabolomic Analysis

**Figure 7:**
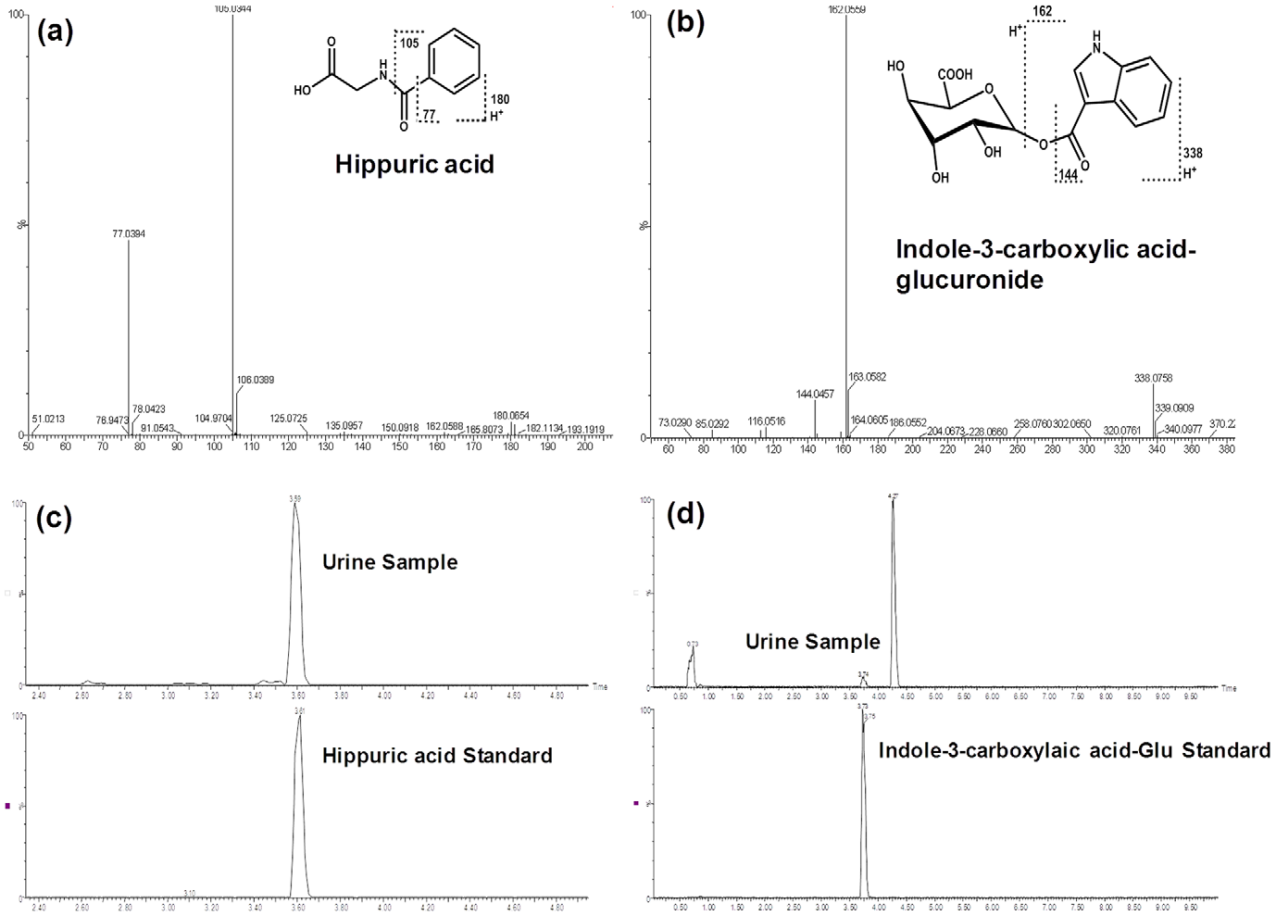
Oatp1a1-null mice had higher hippuric acid, but lower indole-3-carboxylic acid-glucuronide in urine than WT mice. Structural elucidations were performed based on accurate mass measurement and MS/MS fragmentations of hippuric acid (a) and indole-3-carboxylic acid-glucuronide (b) in urine of WT and Oatp1a1-null mice. By comparison of the retention time in the same MS/MS chromatograph window between authentic standards and urine samples, hippuric acid (c) and indole-3-carboxylic acid-G (d) were confirmed. The authentic standard indole-3-carboxylic acid-glucuronide (indole-3-carboxylic acid-G) was enzymatically synthesized from indole-3-carboxylic acid.

The gut microbiota interact extensively with the host through metabolic exchange and co-metabolism of substrates. To investigate the effects of the altered intestinal bacteria on host metabolism, we collected the urine of WT and Oatp1a1-null mice and performed metabolomic analysis using UPLC-TOF-MS. Consistent with a previous study [20], urinary metabonomic analysis in the present study also revealed a distinct separation between WT and Oatp1a1-null mice (Fig. S1). Chemical structural elucidations were performed based on accurate mass measurement and MS/MS fragmentations. By comparison of the retention time in the same MS/MS chromatograph window between authentic standards and urine samples, we identified that Oatp1a1-null mice had higher hippuric acid, but lower indole-3-carboxylic acid-glucuronide (indole-3-carboxylic acid-G) in urine than WT mice (Fig. 7). In addition, Oatp1a1-null mice had lower glucuronides of daidzein and *O*-desmethylangolensin in urine than WT mice (Fig. S2 and S3).

## Discussion

The role of OATP/Oatps in transporting a wide range of structurally unrelated xenobiotics, including numerous drugs and toxins, has drawn the attention of scientists regarding the drug-drug or food-drug interactions that might occur at the transporter level. However, due to the lack of specific inhibitors, the physiological roles of the OATP/Oatp family of transporters still remain unclear. In the present study, we utilized Oatp1a1-null mice and illustrated that this transporter contributes to the regulation of intestinal bacteria population.

The human intestine provides residence to 1×10^13^ bacteria, which is ten-fold higher than the total number of cells in the human body (1×10^12^), and contains a genome with 100-fold more genes than that of humans [24], [25]. These bacteria represent an integral component of the human body, and a dynamic contributor to systems biology. Numerous toxicants have been shown to alter the urinary excretion of bacteria-related metabolites [26]. However, there are limited data regarding the altered intestinal bacteria in the genetically modified animals. In the present study, Oatp1a1-null mice were shown to have a markedly different profile of intestinal bacteria, which resulted in the altered urinary excretion of bacteria-formed metabolites such as hippuric acid and indole-3-carboxylic acid [27], [28]. The novelty of the present finding that loss of Oatp1a1 function leads to alterations in intestinal bacteria is indicative of the complex and relatively unexplored relationship between intestinal bacteria and other organs such as liver.

Intestinal bacteria play a critical role in the metabolism and circulation of BAs. Table S1 and S2 summarized the concentrations or composition of BAs in tissues or contents of WT and Oatp1a1-null mice. Oatp1a1-null mice had similar bile flow (data not shown) and BA concentrations in the bile as WT mice (Fig. 3c and 3d), suggesting that lack of Oatp1a1 does not alter the amounts of BAs entering the small intestine. However, due to the increase in small intestinal bacteria, conjugated BAs (TMCA and TUDCA) decreased, whereas unconjugated BAs (CA, MDCA, HDCA, UDCA, CDCA, and DCA) increased in the small intestinal contents of Oatp1a1-null mice (Fig. 4a and 4b). Consistently, Oatp1a1-null mice also had a markedly different BA profile in the feces, with a decrease in conjugated primary BAs (taurine-, glycine-, and sulfate-conjugated BAs) and an increase in secondary BAs that are produced by 7-dehydroxylation, oxidation, and epimerization (Fig. 2). Unlike other secondary BAs, both TUDCA and UDCA were decreased in the feces of Oatp1a1-null mice (Fig. 2). This may be due to the decrease in *C. absonum*, which expresses both 7α- and 7β-HSDHs, and thus is able to epimerize CDCA to UDCA [7], [8]. Despite the marked changes in BA composition, Oatp1a1-null mice had a similar fecal excretion of total BAs as WT mice (data not shown), consistent with the similar biliary excretion of BAs into the intestine.

Emerging evidence suggests a strong interaction between gut microbiota and human health [29]. Overgrowth of intestinal bacteria is a well-known phenomenon in gastroenterology and pathology [30], [31]. However, the causative mechanisms are not known. The present study provides evidence that the overgrowth of intestinal bacteria can be the result of dysfunction of the liver and bile excreting machineries. For example, overgrowth of Klebsiella frequently gives rise to severe diseases such as septicemia, pneumonia, and soft tissue infection [32]. The present study demonstrates a potential role of liver transporters and bile acids in the homeostasis of Klebsiella. Alterations in intestinal bacteria can also be a contributing factor to the pathophysiology of obesity. The intestinal microbiota in both humans and mice consist mainly of *Bacteroidetes* and *Firmicutes* phyla [3], [4]. The relative proportion of *Bacteroidetes* is decreased in obese people, and increased when these obese people change to a low-calorie diet [33]. Similarly, genetically obese (ob/ob) mice have a 50% reduction in the abundance of *Bacteroidetes*, in the cecal contents [33]. Interestingly, the mRNA and protein expression of Oatp1a1 in livers of obese (ob/ob) mice were diminished to <5% and <15%, respectively, of that in WT mice [34]. In addition, hepatic Oatp1a1 is also suppressed by a high-fat diet in rats [35]. In contrast to ob/ob mice, the present study shows that Oatp1a1-null mice have a 200% increase of *Bacteroidetes*, and a 30% reduction of *Firmicutes* in the large intestine. Thus, there is evidence to suggest that Oatp1a1 expression is altered in the face of metabolic disease such as obesity, and that the function of this transporter is critical for the homeostasis of intestinal bacteria populations.

FXR has been shown to play an important role in preventing bacterial overgrowth and maintaining the integrity of the intestinal epithelium. For example, administration of GW4064, a FXR agonist, blocks bacterial overgrowth and translocation in ilea and ceca of BDL mice [36]. In ileum, the majority of intestinal BAs are absorbed in their conjugated forms via BA transporters, namely the apical sodium dependent bile acid transporter (Asbt) and the organic solute transporter α/β (Ostα/β). Lack of Oatp1a1 decreases the concentrations of conjugated BAs in ilea, and thus may decrease the influx of BAs into ileal enterocytes, resulting in decreased FXR activation. Although lack of Oatp1a1 had little effect on ileal FXR mRNA expression, it significantly decreased the mRNA expression of ileal SHP, a target gene of FXR (Figure 6). In addition, both ileal Fgf15 and hepatic Fgfr4 mRNA expression tend to decrease in Oatp1a1-null mice. This indicates that Oatp1a1-null mice may have a decreased BA-mediated FXR activation in the ilea, which may contribute to the overgrowth of intestinal bacteria. However, it remains unclear how lack of Oatp1a1 alters the intestinal bacteria. It is also possible that lack of Oatp1a1 alters the disposition of some endogenous substrates, other than BAs, which are important in maintaining normal intestinal functions.

In summary, the present study provides a new perspective on the *in vivo* functions of OATPs/Oatps, which are extensively engaged in drug absorption, distribution, and elimination. The alteration of OATP expression and activities can affect the plasma concentration of drugs, thereby significantly influencing drug toxicities, therapeutic efficacies, and drug-drug interactions [37]. The present study suggests that inhibition of Oatp1a1 in mice may result in an overgrowth of intestinal bacteria and thereby an increase of secondary BAs in serum and intestinal contents. The present study also suggests a potential role of OATPs/Oatps in nutrition and obesity. For example, loss of Oatp1a1 function alters urinary excretion of daidzein and its bacteria-mediated metabolite *O*-desmethylangolensin, which have been shown to have beneficial effects on obesity, hypertension, cholesterol, and glucose levels in animals and humans [38]. Therefore, the effects of OATPs/Oatps on intestinal bacteria, BA metabolism, and host metabolomics should be considered when studying drug-induced liver injuries and drug-drug interactions.

## Materials and Methods

### Chemicals and Reagents

BA standards were purchased from Steraloids, Inc. (Newport, Rhode Island) and Sigma-Aldrich (St Louis, MO). Turocholate-7-sulfate was kindly provided by Dr Alan F Hofmann (University of California-San Diego).

### Animals and Breeding

All animal studies were approved by the Institutional Animal Care and Use Committee at the University of Kansas Medical Center. Eight-week-old adult male C57BL/6 mice were purchased from Charles River Laboratories Inc. (Wilmington, MA). Oatp1a1-null mice were bred to homozygosity on the C57BL/6 background as described previously [20]. All mice were housed in an American Animal Associations Laboratory Animal Care (AAALAC) accredited facility with a 12∶12 hr light∶dark cycle and provided chow (Teklad Rodent Diet #8604, Harlan Teklad, Madison, WI) and water *ad libitum*.

### Sample Collection

Age-matched male WT and Oatp1a1-null mice (n = 6/group) were anesthetized i.p. using ketamine (100 mg/kg)/midazolam (5 mg/kg) and the common bile duct was cannulated with a 30-gauge needle attached to PE-10 tubing. Bile was collected from the cannula for 2 hrs at 15-min intervals. Another set of mice (n = 5/group) were anesthetized, blood was collected by orbital bleeding, and serum was obtained by centrifuging blood at 6,000 *g* for 15 min. Livers with gallbladders removed were harvested from the same animals, washed, frozen in liquid nitrogen, and stored at −80°C. Small intestine, cecum, and large intestine were divided, cut open, and vortexed in 3 ml of saline to collect contents, respectively. The three small intestine segments, namely duodenum, jejunum, and ileum, as well as cecum and large intestine, were stored separately at −80°C.

### BA Extraction from Intestinal Contents

To extract BAs from mouse intestinal contents, preliminary experiments were performed to optimize BA extraction. Briefly, intestinal contents were mixed with 100 µl of internal standards (^2^H_4_-GCDCA and ^2^H_4_-CDCA) and centrifuged at 12,000 *g* for 10 min to collect the supernatant. The pellet was extracted with 3 ml of methanol twice. The mixture was centrifuged at 12,000 *g* for 20 min to collect the supernatants. The three supernatants were pooled, evaporated under vacuum, and reconstituted in 1 ml of 50% methanol.

### BA Extraction from Feces

Age-matched male WT and Oatp1a1-null mice (n = 9/group) were acclimated to metabolic cages (housed individually), and feces were collected on ice over a 24-hr period. Mouse feces were dried under vacuum, and ground to a powder. Fifty mg of feces were mixed with BA internal standards and 3 ml of methanol was added. After shaking for 1 hr at room temperature, the mixture was centrifuged at 20,000 *g* for 10 min to collect the supernatant. The pellet was extracted with another 2 ml of methanol. The two supernatants were pooled, evaporated under vacuum, and reconstituted in 100 µl of 50% methanol.

### BA Quantification

BA concentrations were quantified by ultra performance liquid chromatography-tandem mass spectrometry (UPLC-MS/MS) [39]. BA stock solutions were diluted with 50% methanol and spiked with internal standards (^2^H_4_-GCDCA and ^2^H_4_-CDCA) to construct standard curves between 5 and 20,000 ng/ml. All standard curves were constructed using a 1/concentration^2^ weighted quadratic regression, and the correlation coefficient (r^2^) for all BAs was above 0.99. The limit of detection (signal/noise ratio = 3) for the various BAs was in the range of 5–10 ng/ml, which equals 0.01–0.02 nmol/ml.

### Bacterial DNA Extraction

Age-matched male WT and Oatp1a1-null mice (n = 6/group) were anesthetized, and intestinal contents were collected in phosphate buffered saline containing 10 mM dithiothreitol (DTT), and centrifuged at 20,000 *g* for 30 min at 4°C. Total genomic bacterial DNA was extracted from the pellet using QIAmp DNA® stool kit (Qiagen, Valencia, CA) following their instructions. The integrity, concentration, and quality of the total DNA were assessed by agarose gel electrophoresis, and determined by absorption at A_260_, and A_260_ to A_280_ ratio, respectively. DNA solutions were stored at −20°C until further analysis.

### Bacterial Quantification

The bacteria evaluated in the present study were chosen based on a previous report on the major intestinal microflora in mice [4]. Because the majority of the bacteria residing in the intestine were unculturable, the bacterial seuqneces are defined as the closest known relative in the phylogenic tree [4]. A strategy based on quantification of the 16 S rDNA gene by branched DNA (bDNA) assay was utilized to compare the composition of intestinal microbiota of WT and Oatp1a1-null mice. The 16 S rDNA gene assay was performed using Quantigene 2.0 Reagent System (Panomics/Affymetrix, Fremont, CA) according to the manufacturer's protocol. Briefly, the oligonucleotide probes targeted against the 16 S rDNA gene sequences of various bacteria were manufactured by Panomics/Affymetrix (Fremont, CA). Accession numbers for the corresponding bacteria are given in Table 1. The catalog # of each bDNA probe can be reached at www.panomics.com. Our dose-response study determined that the optimal DNA input is 20 ng (Fig. S4). Thus, total bacterial DNA (1 ng/µl, 20 µl) was added to each well containing 80 µl of lysis buffer containing blocking reagent and each probe set. Sample DNA was allowed to hybridize to each probe set overnight at 55°C. Subsequently, the plate was washed with washing buffer three times. Samples were hybridized with the amplification reagent (100 µl/well) in the amplifier/label probe buffer for 1 h at 55°C. The plate was washed 3 times with wash buffer. Label probe diluted in amplifier/label probe buffer was added to each well (100 µl/well), and the alkaline phosphatase-conjugated label probe was allowed to hybridize to the bDNA-DNA complex for 1 h at 50°C. The plate was washed with wash buffer three times. The enzyme reaction was triggered by the addition of substrate solution (100 µl/well) and incubated for 5 min. The resulting luminescence was quantified using a luminometer set at an integration time of 0.2 sec.

**Table 1:** Gene accession numbers of the 16 s rRNA gene assessed in this study.

	Bacterial substrains	NCBI Accession #.
1	*Acetibibrio cellulosolvens*	AJ418058
2	*Bacillus mycoides*	AJ308388
3	*Bacteroid distasonis (i)*	AJ400243
4	*Bacteroid distasonis (ii)*	AJ400242
5	*Bacteroid distasonis (iii)*	AJ400241
6	*Bacteroid distasonis (iv)*	AJ400234
7	*Bacteroid forsythus (i)*	AJ400249
8	*Bacteroid forsythus (ii)*	AJ400236
9	*Bacteroid vulgatus*	AJ400246
10	*Bacteroides distasonis (v)*	AJ400254
11	*Bacteroids acidofaciens*	AJ400252
12	*Bacteroids sp. ASF519*	AJ400245
13	*Bifidobacterium animalis*	AB027536
14	*Candidatus xiphinematobactor*	AJ400275
15	*Clostridium absonum*	X77842
16	*Clostridium celerecrescence (i)*	AJ400265
17	*Clostridium celerecrescens (ii)*	AJ400256
18	*Clostridium clostridiiformes*	AJ400247
19	*Clostridium fusiformis*	AJ400260
20	*Clostridium methylpentosum*	AJ400237
21	*Clostridium perfringens*	FJ384389
22	*Clostridium polysaccharolyticum*	AJ400272
23	*Clostridium scindens*	AY878326
24	*Clostridium sp*	AJ400251
25	*Clostridium sp. ASF502 (i)*	AJ400255
26	*Clostridium sp. ASF502 (ii)*	AJ308396
27	*Clostridium sp. ASF502 (iii)*	AJ400261
28	*Desulfovibrio sp.*	AJ308394
29	*Eubacterium desmolans*	AJ400270
30	*Eubacterium limosum*	AB298910
31	*Helicobactor sp.*	AJ308389
32	*Klebsiella granulomatis*	EU333881
33	*Lactobacillus acidophilus (i)*	AJ400238
34	*Lactobacillus murinus*	AJ308393
35	*Lactobacillus reuteri*	AJ308392
36	*Lactobacillus salivarius*	FJ378897
37	*Lactobacillus sp. ASF519*	AJ308390
38	*Lactobaciluus acidophillus (ii)*	AJ300391
39	*Lactococcus lactis*	FJ378886
40	*Porphyromonas sp. (i)*	AJ400264
41	*Porphyromonas sp. (ii)*	AJ400235
42	*Prevotella sp. (i)*	AJ400267
43	*Prevotella sp. (ii)*	AJ400266
44	*Ralstonia sp.*	AJ308395
45	*Ruminococcus gnavus*	AJ308386
46	*Ruminococcus schinkii*	AJ400250
47	*SFB*	AJ308387
48	*Streptococcus gordnii*	EU156758
49	*TM7 phylum sp*	AJ400239

### Total RNA Isolation

Total RNA was isolated using RNA-Bee reagent (Tel-Test Inc., Friendswood, TX) according to the manufacturer's protocol. Total RNA concentrations were quantified spectrophotometrically at 260 nm. Integrity of RNA samples was determined by formaldehyde-agarose gel electrophoresis with visualization by ethidium bromide fluorescence under ultraviolet light.

### Multiplex Suspension Array

The mRNA expression was quantified by mutiplex suspension array (Panomics/Affymetrix, Fremont, CA). Individual gene accession numbers can be accessed at www.panomics.com (sets #21021 and #21151). Samples were analyzed using a Bio-Plex 200 System Array reader with Luminex 100 xMAP technology, and the data were acquired using Bio-Plex Data Manager version 5.0 (Bio-Rad, Hercules, CA). Assays were performed according to the manufacture's protocol. mRNA data were normalized to Gapdh mRNA and presented as relative light units (RLUs).

### Metabolomic analysis of urine

Urinary samples were prepared by mixing 40 µl of urine with 160 µl of 50% acetonitrile and centrifuged at 20,000 g for 10 min. A 100 mm×2.1 mm UPLC BEH C18 column (Waters) was used for metabolite separation. The flow rate of the mobile phase was 0.3 ml/min with a gradient ranging from 2 to 98% aqueous acetonitrile containing 0.1% formic acid in a 10-min run. TOF-MS was operated in both positive and negative modes with electrospray ionization. The source temperature and desolvation temperature were set at 120 and 350°C, respectively. Nitrogen was applied as the cone gas (10 L/h) and desolvation gas (700 L/h), and argon as the collision gas. TOF-MS was calibrated with [Glu1]-fibrinopeptide and monitored by the intermittent injection of lock mass leucine enkephalin in real time. The capillary voltage and the cone voltage were set at 3.5 kV and 35 V in the positive ion mode. Screening and identification of major metabolites were performed by using MarkerLynx software (Waters) based on accurate mass measurement (mass errors less than 10 ppm). Mass chromatograms and mass spectra were acquired by MassLynx software in centroid format from 50 to 1000 m/z. Centroid and integrated mass chromatographic data were processed by MarkerLynx software to generate a multivariate data matrix. Principal component analysis (PCA) and orthogonal projection to latent structures-discriminant analysis (OPLS-DA) were conducted on Pareto-scaled data. The corresponding data matrices were then exported into SIMCA-P+ (version 12; Umetrics, Kinnelon, NJ) for multivariate data analysis.

### Statistical Analysis

Data are expressed as mean ± S.E. (n = 5–6). Differences between two mean values were tested for statistical significance (*p*<0.05) by the two-tailed Student's t-test.

## Supporting Information

Figure S1 **Metabonomic analysis of WT and Oatp1a1-null mouse urine.** Urine of two-month old male WT and Oatp1a1-null mice (n = 9) were collected for analysis. (a) Separation of WT and Oatp1a1-null mouse urine in a PCA score plot with operation of the TOF-MS in the positive mode. The t [1] and t [2] values represent the score of each sample in principal component 1 and 2, respectively. (b) Loading *S*-plot generated by OPLS-DA analysis of metabonome in urine of Oatp1a1-null mice with the operation of the TOF-MS in the positive mode. The *X*-axis is a measure of the relative abundance of ions and the *Y*-axis is a measure of the correlation of each ion to the model. These loading plots represent the relationship between variables (ions) in relation to the first and second components present in the PCA score plot. (c) Separation of WT and Oatp1a1-null mouse urine in a PCA score plot with operation of TOF-MS in the negative mode. (d) Loading *S*-plot generated by OPLS-DA analysis of metabonome in urine of Oatp1a1-null mice with the operation of TOF-MS in the negative mode.
(TIF)

Figure S2 **Oatp1a1-null mice had lower glucuronides of daidzein and** ***O*** **-desmethylangolensin in urine than WT mice.** Structural elucidations were performed based on accurate mass measurement (mass errors less than 10 ppm) and MS/MS fragmentations of glucuronidated-daidzein (a) and glucuronidated-*O*-desmethylangolensin (b) in urine of WT and Oatp1a1-null mice.
(TIF)

Figure S3 **Confirmation of glucuronidated daidzein and daidzein in urine of WT and Oatp1a1-null mice.** The authentic standard daidzein glucuronides were enzymatically synthesized from daidzein. The enzymatic reaction of daidzein resulted in three glucuronidated-daidzeins, suggesting that daidzein can be glucuronidated at different positions. There is one peak with the same retention time as one of glucuronidated-daidzeins in the urine samples (a). In addition, there are four peaks with the same molecular weight as daidzein in the urine samples, one of which was confirmed as daidzein by the authentic standard (b).
(TIF)

Figure S4 **The effect of DNA input on luminescence of selected bacterial bDNA probes during bacterial quantification.** Total bacterial DNA was added to each well containing 80 µl of lysis buffer containing blocking reagent and each probe set. Sample DNA was allowed to hybridize to each probe set overnight at 55°C. Subsequently, the plate was washed with washing buffer three times. Samples were hybridized with the amplification reagent (100 µl/well) in the amplifier/label probe buffer for 1 h at 55°C. The plate was washed 3 times with wash buffer. Label probe diluted in amplifier/label probe buffer was added to each well (100 µl/well), and the alkaline phosphatase-conjugated label probe was allowed to hybridize to the bDNA-DNA complex for 1 h at 50°C. The plate was washed with wash buffer three times. The enzyme reaction was triggered by the addition of substrate solution (100 µl/well) and incubated for 5 min. The resulting luminescence was quantified using a luminometer set at an integration time of 0.2 sec.
(TIF)

Table S1 Concentrations or composition of individual BAs in tissues or contents of WT and Oatp1a1-null mice. The concentrations of BAs in male WT and Oatp1a1-null mice (n = 5/group) were analyzed using ultra performance liquid chromatography-tandem mass spectrometry (UPLC-MS/MS). All data are expressed as mean ± S.E. of five mice in each group. *, statistically significant difference between WT and Oatp1a1-null mice (*p*<0.05).
(DOC)

Table S2 Calculated secondary BAs and unconjugated BAs in tissues or contents of WT and Oatp1a1-null mice. All data are expressed as mean ± S.E. of five mice in each group. *, statistically significant difference between WT and Oatp1a1-null mice (*p*<0.05).
(DOC)
