# Supplementary figures and images for: Dysfunction of Organic Anion Transporting Polypeptide 1a1 Alters Intestinal Bacteria and Bile Acid Metabolism in Mice
Source: PLoS One. 2012 Apr 4;7(4):e34522. doi: 10.1371/journal.pone.0034522 (PMC3319588; doi:10.1371/journal.pone.0034522)

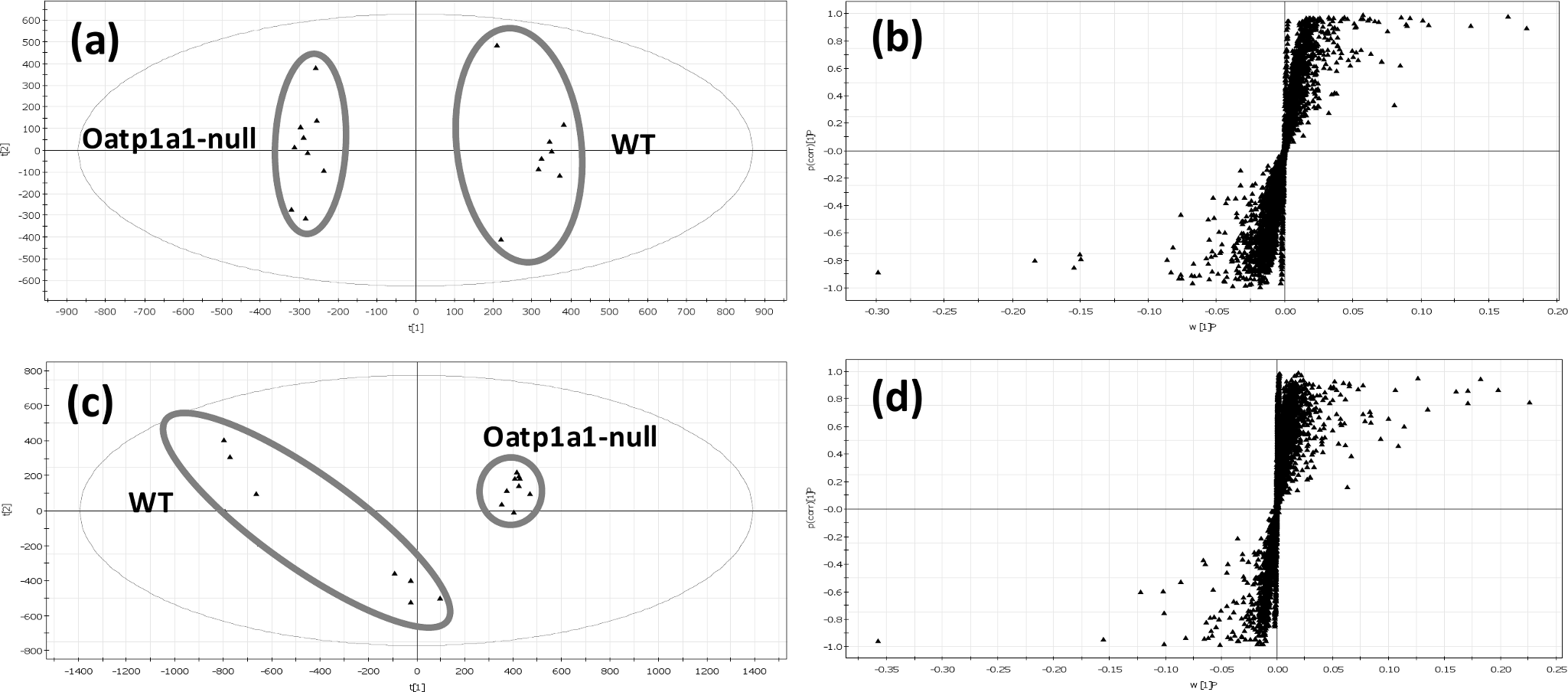

Supplement: Figure S1 — Metabonomic analysis of WT and Oatp1a1-null mouse urine. Urine of two-month old male WT and Oatp1a1-null mice (n = 9) were collected for analysis. (a) Separation of WT and Oatp1a1-null mouse urine in a PCA score plot with operation of the TOF-MS in the positive mode. The t [1] and t [2] values represent the score of each sample in principal component 1 and 2, respectively. (b) Loading S-plot generated by OPLS-DA analysis of metabonome in urine of Oatp1a1-null mice with the operation of the TOF-MS in the positive mode. The X-axis is a measure of the relative abundance of ions and the Y-axis is a measure of the correlation of each ion to the model. These loading plots represent the relationship between variables (ions) in relation to the first and second components present in the PCA score plot. (c) Separation of WT and Oatp1a1-null mouse urine in a PCA score plot with operation of TOF-MS in the negative mode. (d) Loading S-plot generated by OPLS-DA analysis of metabonome in urine of Oatp1a1-null mice with the operation of TOF-MS in the negative mode. (TIF) [file pone.0034522.s001.tif]

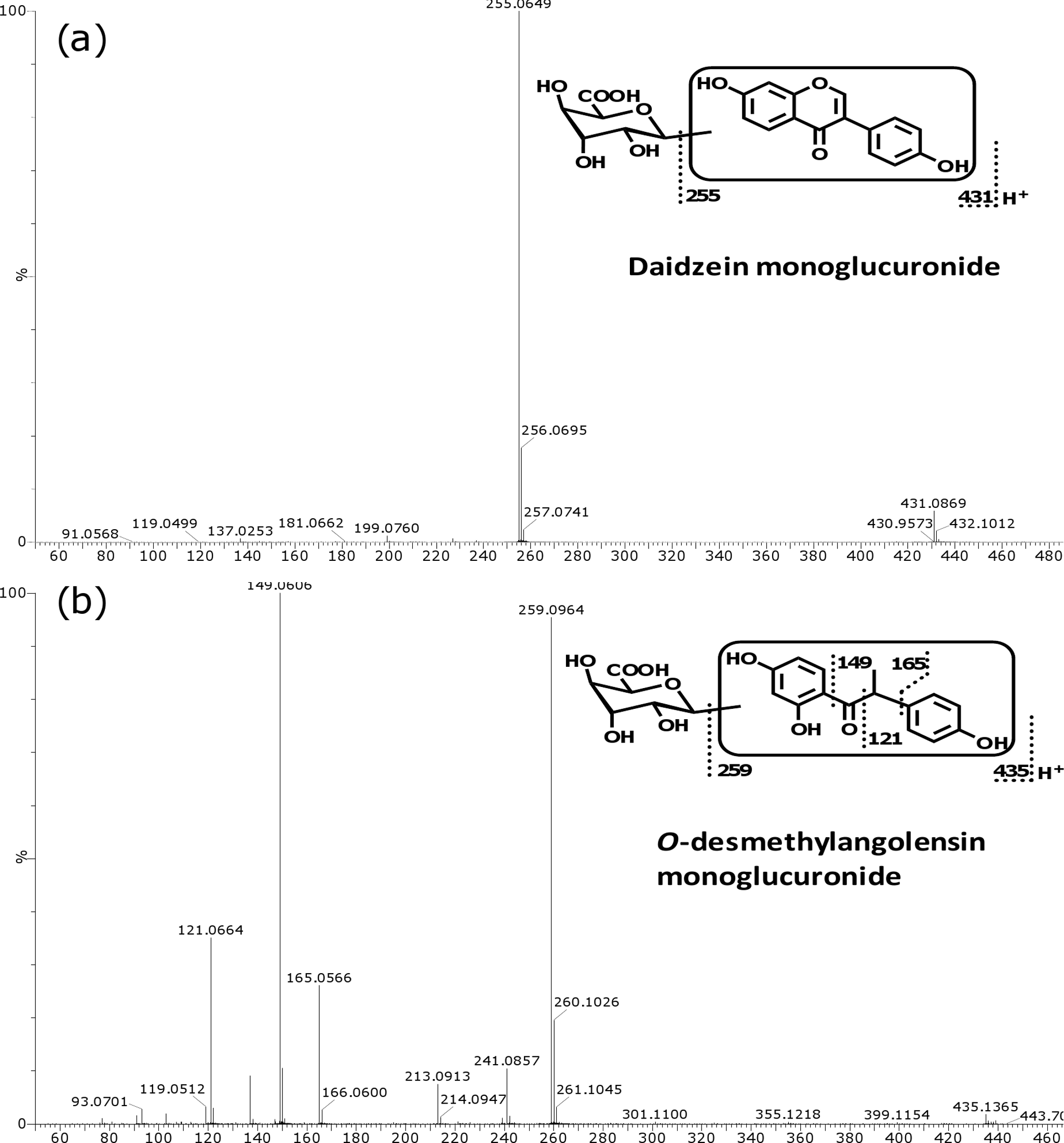

Supplement: Figure S2 — Oatp1a1-null mice had lower glucuronides of daidzein and O -desmethylangolensin in urine than WT mice. Structural elucidations were performed based on accurate mass measurement (mass errors less than 10 ppm) and MS/MS fragmentations of glucuronidated-daidzein (a) and glucuronidated-O-desmethylangolensin (b) in urine of WT and Oatp1a1-null mice. (TIF) [file pone.0034522.s002.tif]

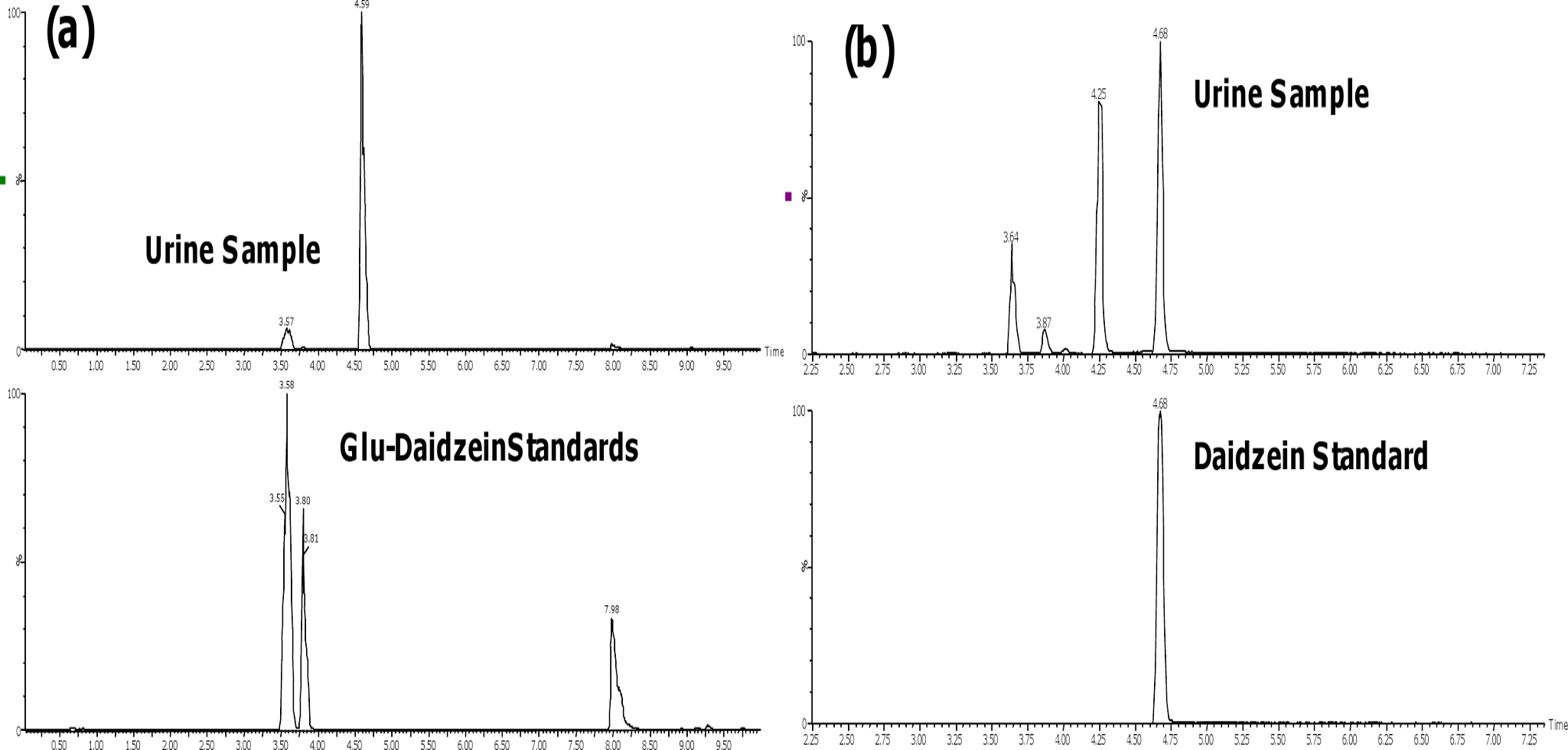

Supplement: Figure S3 — Confirmation of glucuronidated daidzein and daidzein in urine of WT and Oatp1a1-null mice. The authentic standard daidzein glucuronides were enzymatically synthesized from daidzein. The enzymatic reaction of daidzein resulted in three glucuronidated-daidzeins, suggesting that daidzein can be glucuronidated at different positions. There is one peak with the same retention time as one of glucuronidated-daidzeins in the urine samples (a). In addition, there are four peaks with the same molecular weight as daidzein in the urine samples, one of which was confirmed as daidzein by the authentic standard (b). (TIF) [file pone.0034522.s003.tif]

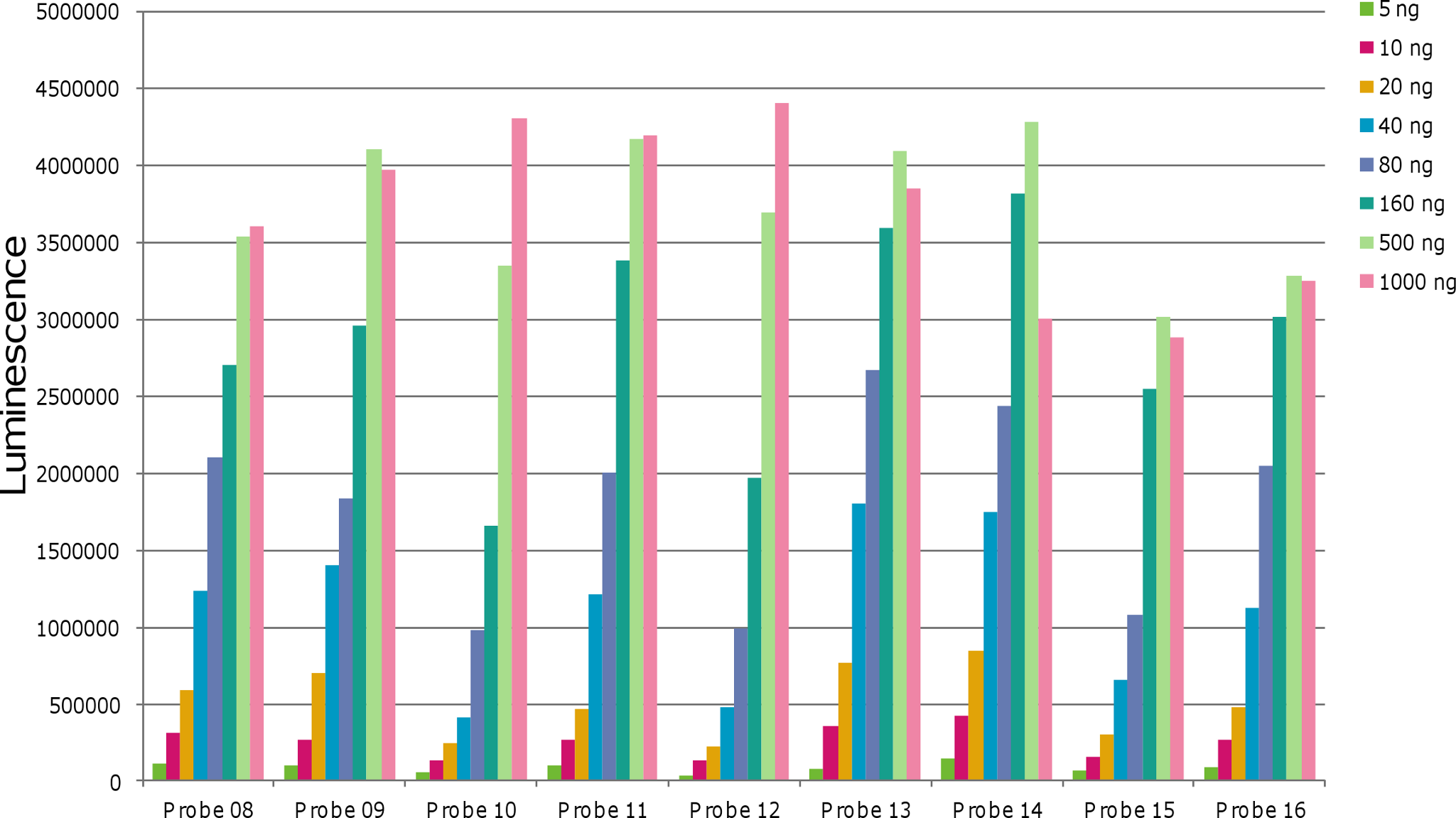

Supplement: Figure S4 — The effect of DNA input on luminescence of selected bacterial bDNA probes during bacterial quantification. Total bacterial DNA was added to each well containing 80 µl of lysis buffer containing blocking reagent and each probe set. Sample DNA was allowed to hybridize to each probe set overnight at 55°C. Subsequently, the plate was washed with washing buffer three times. Samples were hybridized with the amplification reagent (100 µl/well) in the amplifier/label probe buffer for 1 h at 55°C. The plate was washed 3 times with wash buffer. Label probe diluted in amplifier/label probe buffer was added to each well (100 µl/well), and the alkaline phosphatase-conjugated label probe was allowed to hybridize to the bDNA-DNA complex for 1 h at 50°C. The plate was washed with wash buffer three times. The enzyme reaction was triggered by the addition of substrate solution (100 µl/well) and incubated for 5 min. The resulting luminescence was quantified using a luminometer set at an integration time of 0.2 sec. (TIF) [file pone.0034522.s004.tif]
