# Supplementary material for: Dysfunction of Organic Anion Transporting Polypeptide 1a1 Alters Intestinal Bacteria and Bile Acid Metabolism in Mice
Source: PLoS One. 2012 Apr 4;7(4):e34522. doi: 10.1371/journal.pone.0034522 (PMC3319588; doi:10.1371/journal.pone.0034522)
Supplement: Table S1 — Concentrations or composition of individual BAs in tissues or contents of WT and Oatp1a1-null mice. The concentrations of BAs in male WT and Oatp1a1-null mice (n = 5/group) were analyzed using ultra performance liquid chromatography-tandem mass spectrometry (UPLC-MS/MS). All data are expressed as mean ± S.E. of five mice in each group. *, statistically significant difference between WT and Oatp1a1-null mice (p<0.05). (DOC) [file pone.0034522.s005.doc]

**Table S1:** Concentrations or composition of individual BAs in tissues or contents of WT and Oatp1a1-null mice.

| **Liver (nmol/g)** | | | **Bile (µmol/ml)** | | | **Small intestinal content (%)** | | | **Large intestinal content (%)** | | | **Feces (nmol/g)** | | |
| --- | --- | --- | --- | --- | --- | --- | --- | --- | --- | --- | --- | --- | --- | --- |
|  | **WT** | **Oatp1a1** |  | **WT** | **Oatp1a1** |  | **WT** | **Oatp1a1** |  | **WT** | **Oatp1a1** |  | **WT** | **Oatp1a1** |
| TMCA | 6.8±2.3 | 5.3±1.8 | TMCA | 6.2±1.0 | 5.7±0.8 | TMCA | 47.7±2.3 | 18.9±4.5* | TMCA | 0.5±0.1 | 0.1±0.0* | TMCA | 296.7±52.9 | 29.1±2.2* |
| TCA | 81.0±14.5 | 77.1±17.0 | TCA | 13.9±2.1 | 13.4±1.7 | TCA | 35.0±3.4 | 28.9±8.0 | TCA | 0.3±0.1 | 0.1±0.0 | TCA | 316.0±56.2 | 73.1±6.1* |
| TMDCA | 0.0±0.0 | 0.0±0.0 | TMDCA | 0.1±0.0 | 0.2±0.0 | TMDCA | 0.1±0.0 | 0.2±0.1 | TMDCA | 0.01±0.00 | 0.01±0.00 | TMDCA | 1.1±0.3 | 3.0±0.4* |
| TUDCA | 0.9±0.2 | 0.8±0.2 | TUDCA | 0.2±0.0 | 0.2±0.0 | TUDCA | 1.1±0.1 | 0.2±0.1* | TUDCA | 0.02±0.00 | 0.01±0.00* | THDCA | 2.1±0.3 | 3.7±0.4* |
| THDCA | 0.1±0.0 | 0.1±0.0 | THDCA | 0.2±0.0 | 0.1±0.0 | THDCA | 0.1±0.1 | 0.2±0.1 | THDCA | 0.01±0.00 | 0.00±0.00 | TUDCA | 27.9±3.7 | 4.3±0.4* |
| TCDCA | 2.0±0.3 | 2.0±0.4 | TCDCA | 0.4±0.0 | 0.3±0.0 | TCDCA | 0.8±0.2 | 0.7±0.2 | TCDCA | 0.02±0.00 | 0.01±0.00 | TCDCA | 27.0±3.3 | 11.6±1.2* |
| TDCA | 2.8±0.6 | 8.4±1.2* | TDCA | 0.7±0.2 | 1.0±0.2 | TDCA | 0.2±0.0 | 0.3±0.2 | TDCA | 0.02±0.01 | 0.02±0.01 | TDCA | 8.1±4.3 | 12.8±1.2* |
| TLCA | 0.1±0.0 | 0.1±0.0 | TLCA | 0.0±0.0 | 0.0±0.0 | TLCA | 0.0±0.0 | 0.0±0.0 | TLCA | 0.0±0.0 | 0.0±0.0 | TLCA | 0.4±0.3 | 0.8±0.1 |
| w/aMCA | 9.8±1.8 | 6.2±1.5 | w/aMCA | 0.1±0.0 | 0.0±0.0 | wMCA | 2.2±1.1 | 2.5±0.7 | wMCA | 38.6±6.9 | 29.1±4.3 | aMCA | 18.7±1.2 | 36.4±4.1* |
| bMCA | 34.7±7.3 | 28.9±7.3 | bMCA | 0.0±0.0 | 0.0±0.0 | bMCA | 4.4±1.6 | 12.8±3.5 | bMCA | 40.2±3.8 | 23.7±4.0* | bMCA | 140.6±10.9 | 93.8±8.5* |
| CA | 4.6±0.9 | 4.3±1.3 | CA | 0.1±0.0 | 0.1±0.1 | CA | 7.5±2.6 | 32.4±8.3* | CA | 2.0±0.4 | 0.8±0.3 | CA | 71.2±7.1 | 24.6±4.6* |
| MDCA | 0.2±0.0 | 0.4±0.0 | CDCA | 0.0±0.0 | 0.0±0.0 | MDCA | 0.0±0.0 | 0.2±0.0* | MDCA | 0.1±0.1 | 0.4±0.1 | MDCA | 1.5±0.1 | 3.5±0.3 |
| UDCA | 1.1±0.2 | 1.2±0.3 |  |  |  | UDCA | 0.2±0.1 | 0.5±0.1* | UDCA | 0.5±0.1 | 0.2±0.0* | UDCA | 4.8±0.5 | 2.0±0.4* |
| HDCA | 0.5±0.1 | 0.6±0.1 |  |  |  | HDCA | 0.1±0.0 | 0.3±0.1* | HDCA | 1.6±0.4 | 2.7±0.6 | HDCA | 1.5±0.5 | 26.7±3.3* |
| CDCA | 1.7±0.2 | 1.2±0.2 |  |  |  | CDCA | 0.3±0.1 | 0.7±0.2* | CDCA | 0.3±0.1 | 0.8±0.2* | CDCA | 6.7±0.6 | 10.4±1.8* |
| DCA | 0.3±0.1 | 0.3±0.1 |  |  |  | DCA | 0.1±0.0 | 1.1±0.3* | DCA | 9.5±1.5 | 39.0±2.5* | DCA | 15.9±8.3 | 215.3±23.3* |
| LCA | 0.0±0.0 | 0.0±0.0 |  |  |  | LCA | 0.0±0.0 | 0.0±0.0 | LCA | 0.7±0.2 | 3.0±0.8* | LCA | 0.8±0.4 | 16.5±2.5* |
|  |  |  |  |  |  |  |  |  |  |  |  | GDCA | 0.1±0.0 | 0.3±0.0* |
|  |  |  |  |  |  |  |  |  |  |  |  | isoDCA | 1.4±0.6 | 18.3±3.2* |
|  |  |  |  |  |  |  |  |  |  |  |  | isoLCA | 0.1±0.0 | 2.1±0.3* |
|  |  |  |  |  |  |  |  |  |  |  |  | dehydroLCA | 0.1±0.1 | 6.5±1.0* |
|  |  |  |  |  |  |  |  |  |  |  |  | 6-oxoLCA | 0.6±0.1 | 4.9±0.5* |
|  |  |  |  |  |  |  |  |  |  |  |  | 7-oxoLCA | 2.5±0.2 | 4.2±0.2* |
|  |  |  |  |  |  |  |  |  |  |  |  | 12-oxoLCA | 14.2±6.1 | 194.4±12.7* |

Note: The concentrations of BAs in male WT and Oatp1a1-null mice (n=5/group) were analyzed using ultra performance liquid chromatography-tandem mass spectrometry (UPLC-MS/MS). All data are expressed as mean ± S.E. of five mice in each group. *, statistically significant difference between WT and Oatp1a1-null mice (*p*<0.05).
