# Supplementary material for: Dysfunction of Organic Anion Transporting Polypeptide 1a1 Alters Intestinal Bacteria and Bile Acid Metabolism in Mice
Source: PLoS One. 2012 Apr 4;7(4):e34522. doi: 10.1371/journal.pone.0034522 (PMC3319588; doi:10.1371/journal.pone.0034522)
Supplement: Table S2 — Calculated secondary BAs and unconjugated BAs in tissues or contents of WT and Oatp1a1-null mice. All data are expressed as mean ± S.E. of five mice in each group. *, statistically significant difference between WT and Oatp1a1-null mice (p<0.05). (DOC) [file pone.0034522.s006.doc]

**Table S2: Calculated secondary BAs and unconjugated BAs in tissues or contents of WT and Oatp1a1-null mice.**

|  | **Liver (nmol/g)** | | **Bile (µmol/ml)** | | **Small intestinal content (%)** | | **Large intestinal content (%)** | | **Feces (nmol/g)** | |
| --- | --- | --- | --- | --- | --- | --- | --- | --- | --- | --- |
|  | **WT** | **Oatp1a1-null** | **WT** | **Oatp1a1-null** | **WT** | **Oatp1a1-null** | **WT** | **Oatp1a1-null** | **WT** | **Oatp1a1-null** |
| **Secondary BAs** | 6.0±1.2 | 12.0±2.1 | 1.3±0.3 | 1.5±0.3 | 4.2±1.5 | 5.5±1.6 | 51.1±9.3 | 74.3±8.3* | 83.1±26.0 | 519.2±50.4* |
| **Unconjugated BAs** | 52.8±10.7 | 43.2±10.9 | 0.3±0.1 | 0.2±0.1 | 14.8±5.5 | 50.4±13.1* | 93.5±13.6 | 99.6±12.8 | 280.6±36.8 | 659.6±66.8* |

Note: All data are expressed as mean ± S.E. of five mice in each group. *, statistically significant difference between WT and Oatp1a1-null mice (*p*<0.05).
